# The Drake Passage opening from an experimental fluid dynamics point of view

**DOI:** 10.1038/s41598-021-99123-0

**Published:** 2021-10-07

**Authors:** Miklós Vincze, Tamás Bozóki, Mátyás Herein, Ion Dan Borcia, Uwe Harlander, Attila Horicsányi, Anita Nyerges, Costanza Rodda, András Pál, József Pálfy

**Affiliations:** 1 von Kármán Laboratory of Environmental Flows, Eötvös Loránd University, Budapest, 1117 Hungary; 2 MTA-ELTE Theoretical Physics Research Group, Budapest, 1117 Hungary; 3 Doctoral School of Environmental Sciences, University of Szeged, Szeged, 6720 Hungary; 4 Institute of Earth Physics and Space Science (ELKH EPSS), Sopron, 9400 Hungary; 5 Department of Statistical Physics and Nonlinear Dynamics, Brandenburg University of Technology Cottbus–Senftenberg, Cottbus, 03046 Germany; 6 Department of Aerodynamics and Fluid Mechanics, Brandenburg University of Technology Cottbus–Senftenberg, Cottbus, 03046 Germany; 7 Department of Geology, Eötvös Loránd University, Budapest, 1117 Hungary; 8 MTA-MTM-ELTE Research Group for Paleontology, 1431 Budapest, Hungary; 9 Laboratoire des Ecoulements Géophysiques et Industriels, Université Grenoble Alpes, CNRS, Grenoble-INP, 38000 Grenoble, France; 10 Konkoly Observatory, Research Centre for Astronomy and Earth Sciences, Eötvös Loránd Research Network (ELKH), 1121 Budapest, Hungary; 11 Institute for Theoretical Physics, Eötvös Loránd University, Budapest, 1117 Hungary

**Keywords:** Physical oceanography, Fluid dynamics, Palaeoceanography, Climate sciences, Ocean sciences

## Abstract

Pronounced global cooling around the Eocene–Oligocene transition (EOT) was a pivotal event in Earth’s climate history, controversially associated with the opening of the Drake Passage. Using a physical laboratory model we revisit the fluid dynamics of this marked reorganization of ocean circulation. Here we show, seemingly contradicting paleoclimate records, that in our experiments opening the pathway yields *higher* values of mean water surface temperature than the “closed” configuration. This mismatch points to the importance of the role ice albedo feedback plays in the investigated EOT-like transition, a component that is not captured in the laboratory model. Our conclusion is supported by numerical simulations performed in a global climate model (GCM) of intermediate complexity, where both “closed” and “open” configurations were explored, with and without active sea ice dynamics. The GCM results indicate that sea surface temperatures would change in the opposite direction following an opening event in the two sea ice dynamics settings, and the results are therefore consistent both with the laboratory experiment (slight warming after opening) and the paleoclimatic data (pronounced cooling after opening). It follows that in the hypothetical case of an initially ice-free Antarctica the continent could have become even warmer after the opening, a scenario not indicated by paleotemperature reconstructions.

## Introduction

The opening of major gateways in the Southern Ocean, namely the Drake Passage and the Tasmanian Seaway around the Eocene–Oligocene boundary ca. 34 Ma, is widely regarded as a key contributor to the thermal isolation of Antarctica (that has reached its polar position already in the Cretaceous period) and the glaciation of the continent^1,2^. The Eocene–Oligocene transition (EOT) is marked by a major climatic change on the global scale, with paleotemperature records indicating a rapid drop of global mean temperature. The EOT also coincides with the largest extinction event in the Cenozoic^3^, and extraterrestrial impact events are also known from this interval^4^.

The opening of the southern seaways enabled the development of the Antarctic Circumpolar Current (ACC) which inhibits warm surface currents to transport heat from the Tropics to the Antarctic continent. The details of the role that the onset of ACC played in the growth of continental-scale Antarctic ice sheet (AIS) are still far from being fully resolved^5^. In their seminal paper DeConto and Pollard^6^ argued that the opening of gateways was merely a secondary factor for AIS development and falling atmospheric CO_2_ level was the main driver of EOT. However, significant gaps and contradictions exist in the available CO_2_ proxy data in the most relevant time interval between 40 and 30 Ma^7^, thus setting a precise timeline of events (i.e. CO_2_ decrease, temperature drop, Drake Passage opening), and hence to establish their causal links remains controversial. Here we revisit this problem with a focus on the climate impact of Drake Passage opening, using a novel combination of a laboratory experiment and numerical simulations.

The present-day ACC is dominantly driven by wind stress and the meridional buoyancy contrast (yielding quasi-geostrophic flow) of the ocean itself, whose sea surface temperature (SST) field exhibits a strong temperature difference of $\sim 10^\circ$C between the latitudes of $70^\circ$S and $35^\circ$S. The enormous material and momentum transport associated with ACC—estimated at 100–150 Sv^8^ making it the largest ocean current—is facilitated by the unique geographical boundary conditions of the Southern Ocean, the only ocean basin on the planet that is not confined by meridional continental boundaries. The “thermal wind balance” facilitated by this flow tends to maintain tilted density interfaces (isopycnals) sloping northward, yielding baroclinic instability in the ACC^9,10^. Analogously to atmospheric weather systems the instability generates ubiquitous eddies in the Southern Ocean, with a typical scale of $\sim$ 100 km. The mass and heat transport associated with these vortices, in return, acts to flatten out the tilted density levels and constitutes the key driving force of poleward mass transfer and overturning circulation in the present-day ACC^11–13^. Blocking this circumpolar zonal pathway with a continental barrier would deviate the flow to form meridional boundary currents which in turn would lead to the buildup of a zonal (east-west) gradient in sea surface temperature and a significantly larger meridional heat transport between the equatorial and polar regions than in the “open” configuration (i.e. without the barrier)^14,15^.

Here we do not aspire to accurately model neither the actual paleoceanographic processes in their vast complexity, nor the present-day Southern Ocean dynamics that cannot be fully understood without considering, e.g., water exchange with the Atlantic and internal mixing. Instead, inspired by the significance of the Drake Passage opening, the present study investigates the underlying physical basics of the heat exchange dynamics in a topologically similar, but radically simplified system. The widespread claim which we intend to critically address is that an opening event resulting in the formation of a circumpolar pathway could, in itself, lead to the thermal isolation (and glaciation) of the polar region and, eventually, the decrease of global temperature, via blocking meridional heatflow. If so, this reasoning would suggest that Antarctica may not have necessarily been covered with a permanent ice sheet before the formation of the circumpolar pathway. It is also possible, however, that due to other reasons the glaciation preceded the Drake Passage opening, and the latter merely accelerated the further cooling. Thus, regarding the physical basics of the problem, the key question we are focusing on is whether or not the topological reorganization of the ocean currents, facilitated by an opening event, is sufficient to yield cooling *per se*. Our experimental and numerical findings suggest that if we do not incorporate sea ice dynamics in our models, approximating the case of an initially ice-free Antarctica, then the expected global cooling does not occur, somewhat contradicting the traditional reasoning. However, if the continent is cold enough, thus sea ice is present, then the opening can indeed enhance further glaciation and cooling. These findings, although admittedly conceptual in nature, may contribute to the better understanding of the timing of the various EOT-related events.

## Laboratory experiments

**Figure 1:**
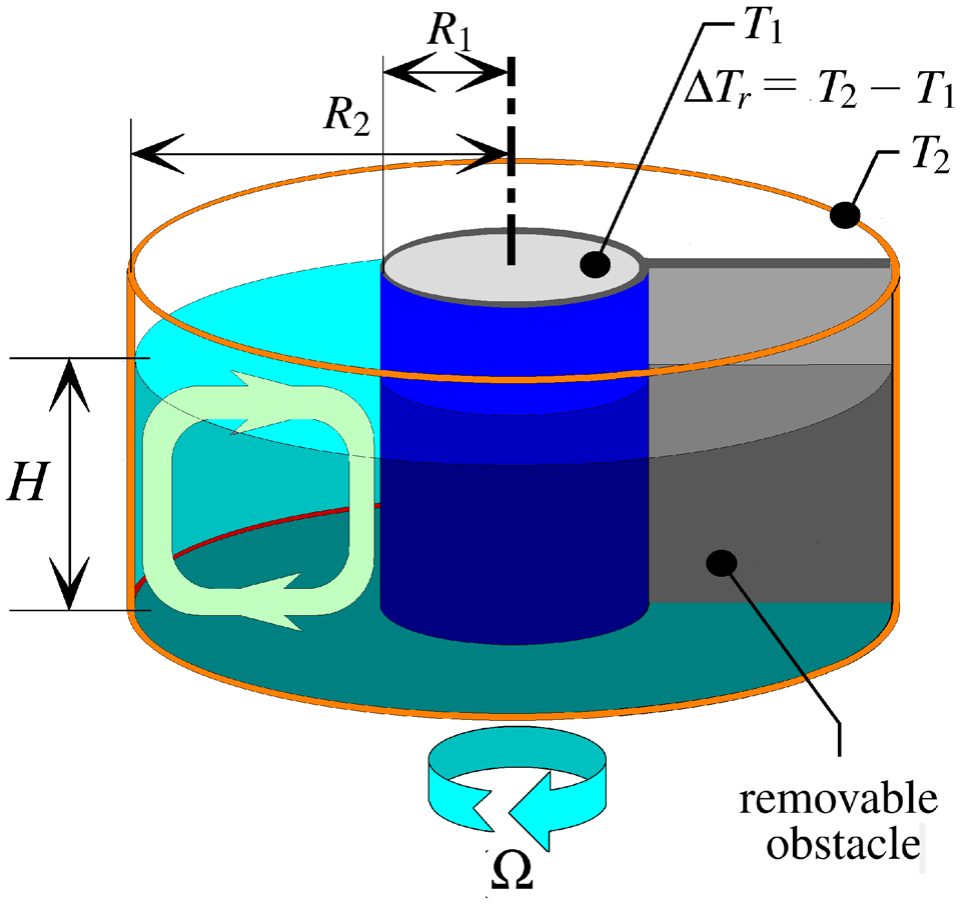
The sketch of the experimental setup. The water body is located in the annular domain between the two coaxial cylinders. The entire tank is rotating around its axis in the clockwise direction at rotation rate $\Omega$—as indicated by the arrow at the bottom—to imitate the Southern Hemisphere configuration. The inner and outer sidewalls are kept at constant cold ($T_1$) and warm ($T_2$) temperature, respectively, maintaining a radial (“meridional”) temperature difference forcing, $\Delta T_r=T_2 - T_1$. The removable vertical obstacle is sketched with gray. The infrared sensor was mounted above the annular gap (not co-rotating), and thus scanned the surface temperature field of the water as it moved underneath. The light green loop (left) depicts the direction of the basic overturning flow in the tank. The figure was created using KolourPaint for Ubuntu linux, Release 20.12.3 (freely available and downloadable at https://apps.kde.org/kolourpaint/).

Due to hydrodynamic similarity certain key aspects of the complexity of such planet-scale flow systems can be modeled rather accurately in relatively simple tabletop-size experiments. The apparatus applied in the present work is one version of a laboratory setup widely used in atmospheric and ocean dynamics^16–19^: the so-called differentially heated rotating annulus, introduced by Raymond Hide in the early 1950s^20^. The setting captures the two most relevant factors in the formation of ACC-like geostrophic jets and baroclinic surface vortices, namely the meridional temperature gradient and planetary rotation (Fig. 1). The applicability of the setting to investigate large-scale ocean circulation in the “closed” Drake Passage configuration has been demonstrated in a recent pilot study^15^.

The layout of the apparatus is sketched in Fig. 1. The tank is mounted on a turntable so that its axis of symmetry coincides with the axis of rotation. The vertical sidewalls of the tank consist of two coaxial cylinders: the cooled inner cylinder (modeling Antarctica) and the heated outer rim, characterised by temperatures $T_1$ and $T_2$, and radii $R_1$ and $R_2$, respectively. The working fluid (water), representing the Southern Ocean, occupies the annular gap between the two cylinders up to level *H*, and therefore experiences a certain “meridional” temperature difference $\Delta T_r=T_2 - T_1$. The top of the tank is not covered to enable the observation of the water surface temperature (WST) field with infrared thermography. The geometrical and dynamical parameters of the setup had to be set to ensure approximate dynamic similarity with the actual Southern Ocean, as discussed in “Methods”. A thin removable vertical insulating barrier reaching through the full water depth represents the Antarctica-South America land bridge (gray wall in Fig. 1).

Each experimental run was conducted as follows. After setting the temperature of the inner and outer sidewalls and the rotation rate to their prescribed values, the experiment was kept running for over ${\sim }3500$ revolutions—a time interval sufficient for quasi-stationary flow patterns to develop—with the barrier in place (“closed” configuration) of which the last ${\sim }1700$ revolution-long leg was later evaluated. Then the barrier was removed by pulling it upward instantaneously, while the rotation rate and the thermal control remained unchanged. In the resulting “open” configuration the data acquisition continued for an additional ${\sim }1700$ revolutions. (More details about the measurement techniques are given in the “Methods” section.)

**Figure 2:**
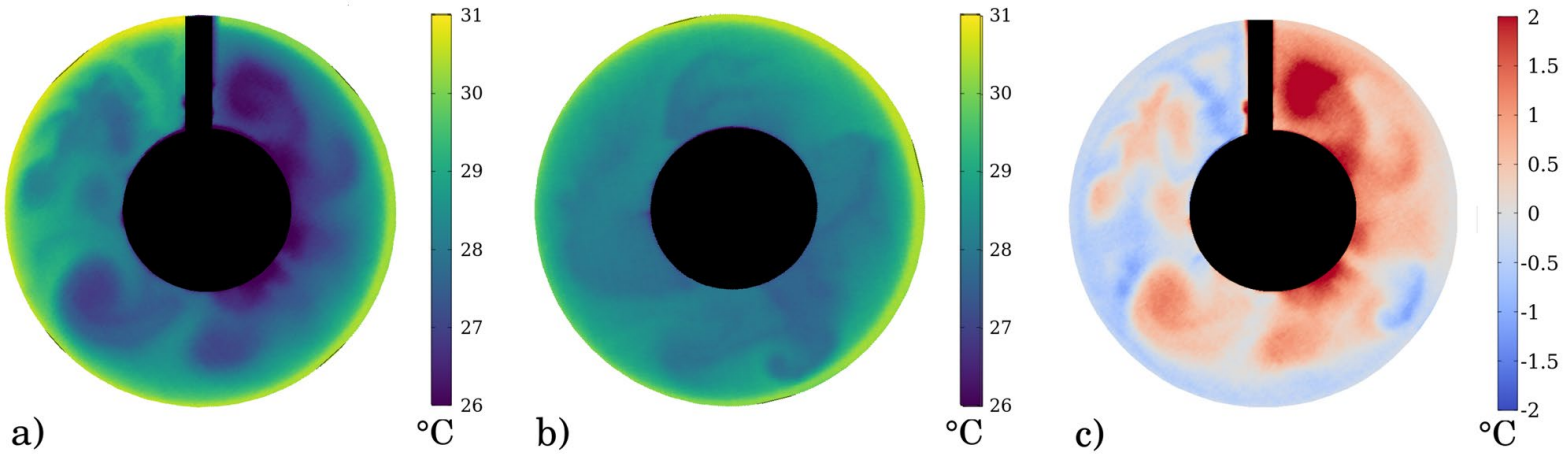
Water surface temperature patterns. Infrared thermographic maps of the water surface temperature (WST) fields in the “closed” (**a**) and “open” (**b**) leg of an experiment, and their differences (“open” minus “closed”, **c**). ($\Omega = 2.0$ rad/s, $\Delta T_r = 11^\circ$C, $H = 5$ cm). The figure was created using Gnuplot version 5.2, Release 5.2.8 (freely available and downloadable at http://www.gnuplot.info/).

Figure 2a shows a typical WST map from a “closed” experiment with a clearly visible zonal temperature gradient and a marked cold anomaly (blue) at the “eastern” side of the barrier (as reported in^15^). In panel (b) the WST field of the “open” configuration subject to the same forcing is presented, exhibiting irregular baroclinic (Rossby) waves and much less pronounced temperature fluctuations (note that the color scales of Fig. 2a and b are identical). Fig. 2c shows the “open”–“closed” differential pattern, showing an overall increase of the WST after the removal of the barrier. Note that this is a difference of two snapshots, not of the time-averaged patterns. For a more detailed analysis of the mean azimuthal WST field in the experiment we refer to our earlier study^15^.

**Figure 3:**
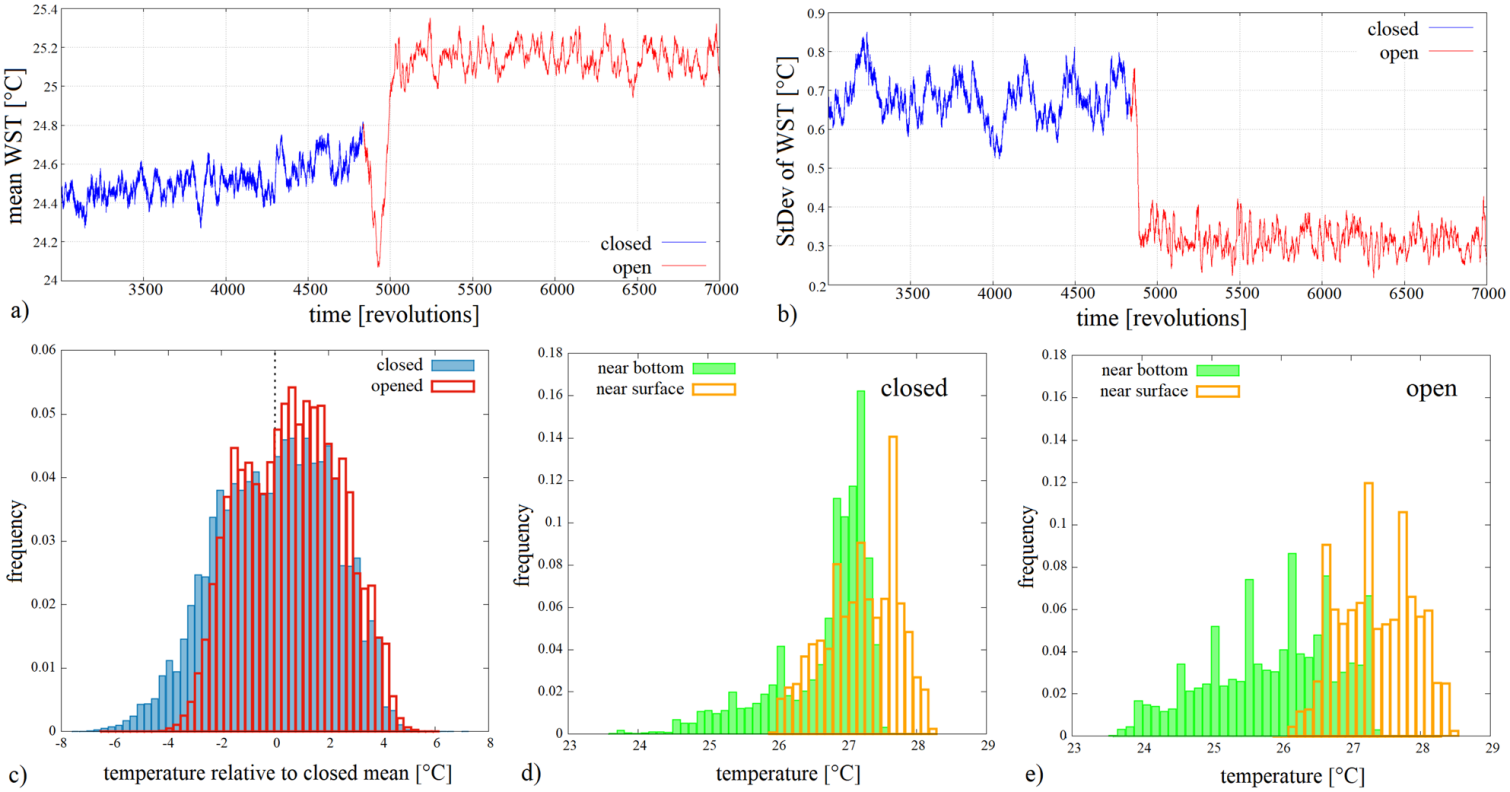
Water surface temperature (WST) distributions before and after the opening. (**a**) The time series of the spatially averaged WST field at each time instant, following a 10-revolution running averaging for smoothing. The change of color (from blue to red) marks the removal of the barrier ($\Omega = 2.0$ rad/s, $\Delta T_r = 18.7^\circ$C). (**b**) The spatio-temporal standard deviation of the fluctuations of the WST field, as calculated with a 10-revolution moving window in the same experiment as in panel (**a**). (**c**) Histograms of the WST field of the entire surface acquired from the data of ${\sim }$1700 revolutions in both the “closed” (blue) and “open” (red) legs of the same experiment as of panels (**a**) and (**b**). (**d**, **e**), Histograms of temperature time series obtained at the same horizontal location via co-rotating temperature sensors placed underneath each other, one close to the surface, at a depth of 6 mm and the other above the bottom by 5 mm from a control experiment (with a total water height of $H = 5$ cm). The near-surface histograms are shown with orange and the near-bottom ones are green. Panel (**d**) represents the “closed” and panel (**e**) the “open” leg of the same experiment ($\Omega = 2.0$ rad/s, $\Delta T_r = 11 ^\circ$C). The figure was created using Gnuplot version 5.2, Release 5.2.8 (freely available and downloadable at http://www.gnuplot.info/).

This abrupt reorganization of the flow is reflected in a typical time series in Figs. 3a and b as well. Panel (a) shows a “meridionally” (i.e. radially) averaged, zonally scanned temperature time series smoothed by a ca. 10-revolution (491-point) running mean. The record, hence, accounts for the temporal development of the spatially averaged WST field. The blue section of the curve corresponds to the “closed” state, whereas the “open” leg of the experiment, exhibiting significantly higher mean temperatures (after a roughly 200-revolution transient phase) is marked with red. The rapid transition is also indicated by the abrupt drop of the running standard deviation of the temperature field (using the same window length) (Fig. 3b). The histograms of Fig. 3c represent the spatial and temporal variability of the WST distribution of the entire water surface before and after the opening event [with the same color coding as in panels (a) and (b)]. Apparently, the opening caused a significant shift of the distribution towards higher temperatures, the disappearance of the cold region at the “eastern” side of the obstacle (cf. Figs. 2a,b) and, as a consequence, of the fat left tail in the “closed” histogram in panel (c).

The histograms of Fig. 3d and e are obtained in a control experiment where the setup was equipped with co-rotating temperature sensors (thermocouples) measuring near-surface and near-bottom water temperature time series at mid-radius, at an azimuthal position angle (“longitude”) of ca. $90^\circ$ from the barrier. Panels (d) and (e) show the measured temperature distributions before and after the opening, respectively. Clearly, the surface (orange) and bottom (green) temperature distributions of the “closed” case largely overlap with each other, whereas in the “open” configuration the surface and bottom histograms shifted toward higher and lower temperatures, respectively, indicating stronger vertical stratification. It is to be noted, however, that the measurements at this single location are not necessarily representative to the zonal average temperature distribution in the “closed” case.

**Figure 4:**
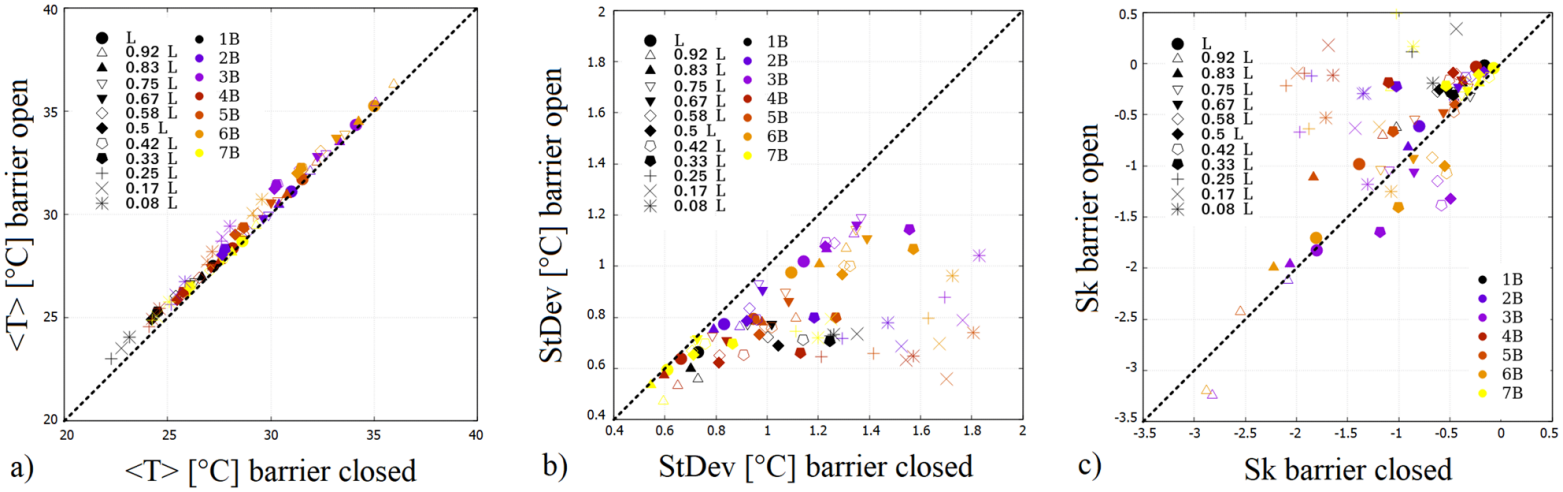
Temperature statistics before and after the opening. Zonal and temporal mean WST (**a**), and the standard deviation (**b**) and skewness (**c**) of the same signals after vs. before the opening. The data are acquired from 7 experiments, which are marked with different colors, and from 12 zonal contours (“latitudes”) shown by different symbols. In the legend, the distance of each “latitude” is given from the inner cylinder (of radius $R_1$) in the units of total gap width $L = R_2 - R_1$ (cf. Fig. 1). The figure was created using Gnuplot version 5.2, Release 5.2.8 (freely available and downloadable at http://www.gnuplot.info/).

**Table 1:** The list of experiments.

run ID	$\langle \Delta T_{r}\rangle$ [$^{\circ }$C]	$Ro_T$
1B	18.7	0.043
2B	22.7	0.052
3B	25.6	0.059
4B	14.6	0.034
5B	17.8	0.041
6B	21.9	0.050
7B	14.6	0.034
1C	11.0	0.045
2C	11.0	0.045

The values of $\langle \Delta T_{r}\rangle$ represent the temporal average temperature differences between the inner and outer walls (transients are omitted). Experiment IDs ending with B and C denote runs conducted in the Budapest and Cottbus tanks, respectively. The value of the Taylor number for the Budapest tank is $Ta_{B}=4.05\times 10^9$ and for the Cottbus tank $Ta_{C} = 8.26\times 10^8$ in all experiments.

The comparison of the mean, standard deviation (StDev) and skewness (Sk) of the first (closed) and second (open) leg of the WST signals from the 12 infrared channels (“latitudes”) in 7 experiments is presented in Figs. 4a,b, and c, respectively. The runs differed in the preset values of the “meridional” temperature contrast $\Delta T_r$ (see “Methods” section). Each symbol represents one channel (“latitude”) of a given experiment, whose radial distance from the sidewall of the inner cylinder is expressed in units of total gapwidth $L = R_2 - R_1$ in the legend. The different runs are distinguished by the coloring of the data points (cf. Table 1). Thus, in panel (a) one data point marks a time-averaged “zonal mean” temperature $\langle T \rangle$ for one “latitude” and one experimental run. The point’s horizontal coordinate represents its value in the closed leg and the vertical coordinate gives its value in the open leg of the experiment. Apparently, all values scatter above the dashed $y = x$ line. Therefore, the zonal and temporal average WST has increased in each run and at all “latitudes” after the removal of the barrier, in concert with the vanishing of the cold surface anomaly. This change is accompanied with a clear decrease of the signals’ standard deviations (Fig. 4b) and—as the histogram of Fig. 3b also demonstrates—the transition from typically left-skewed distributions towards more symmetrical ones (Fig. 4c).

Our fluid dynamical interpretation of the above observations is the following: in the “closed” configuration the barrier facilitates the buildup of a marked azimuthal (zonal) pressure gradient associated with strong radial (meridional) flow which enhances full-depth overturning. According to the “conjecture” of Raymond Hide^14^, in this configuration the radial advective heat transport is independent of rotation rate $\Omega$.

In the “open” case, where a mean zonal pressure gradient would not get established, the advective heat transfer in the meridional direction shows a nontrivial dependence on the rotation rate—or, more precisely, on the thermal Rossby number—of the system. (If the setup was not rotating there would be practically no difference between the two configurations in this respect.) With the increasing angular velocity the radial heat transport steadily decreases, up until the point where the rotation rate is large enough—i.e. the thermal Rossby number is small enough—for the onset of baroclinic instability. Then, with the emergence of eddies, two new ways of meridional heat and mass transport emerge and become increasingly dominant: direct eddy flux, and the perturbation of the zonally symmetric meridional transport by the action of the eddies. These eddy-related contributions compensate for the decreasing transport attributed to Ekman layer transport so that the total meridional heat flow remains almost constant, but still smaller than in the non-rotating (or the “closed”) case. According to a combined numerical and experimental analysis^21^, for a differentially heated rotating annulus within the Rossby number range investigated here, it is expected that these eddy effects are responsible for the largest part (at least 60%) of the total advective meridional heat transport, in analogy with the situation of the present-day Southern Ocean, where overturning is also primarily eddy-driven.

The stronger overturning—characteristic to the “closed” configuration—yields a reduced temperature contrast between the near-surface and deeper domains of working fluid. Hence, in this case, it is reasonable to assume that the average surface temperature is closer to the volumetric average of the whole water body. In the “open” setting, however, the dominantly eddy-driven overturning (turbulent or wave) yields weaker heat transport and, hence, larger vertical temperature gradients (cf. Figs. 3d and e), thus conserving a generally warmer WST field.

To summarize, the sign of the surface temperature shift observed in the experiments is the opposite of the one associated with the actual EOT climate change. We can conclude that the laboratory experiment is not a sufficient representation of the Southern Ocean in this respect. Although the laboratory model captures the basic overturning dynamics of the actual ocean, it lacks important feedback mechanisms, most notably atmospheric and sea ice dynamics. In order to study the combined effect of these components of the climate system and to disentangle the competing feedback processes, we turned to numerical simulations.

## Simulations in a GCM

Our numerical investigations were conducted with a general circulation model or global climate model (GCM) of intermediate complexity, the so-called Planet Simulator (PlaSim), developed at the University of Hamburg^22,23^, the technical basics of which are briefly described in the “Methods” section and further details are given in the Supplementary Material. The model incorporates atmospheric and—when coupled with a large-scale geostrophic (LSG) ocean module^24^—full-depth ocean dynamics.

**Figure 5:**
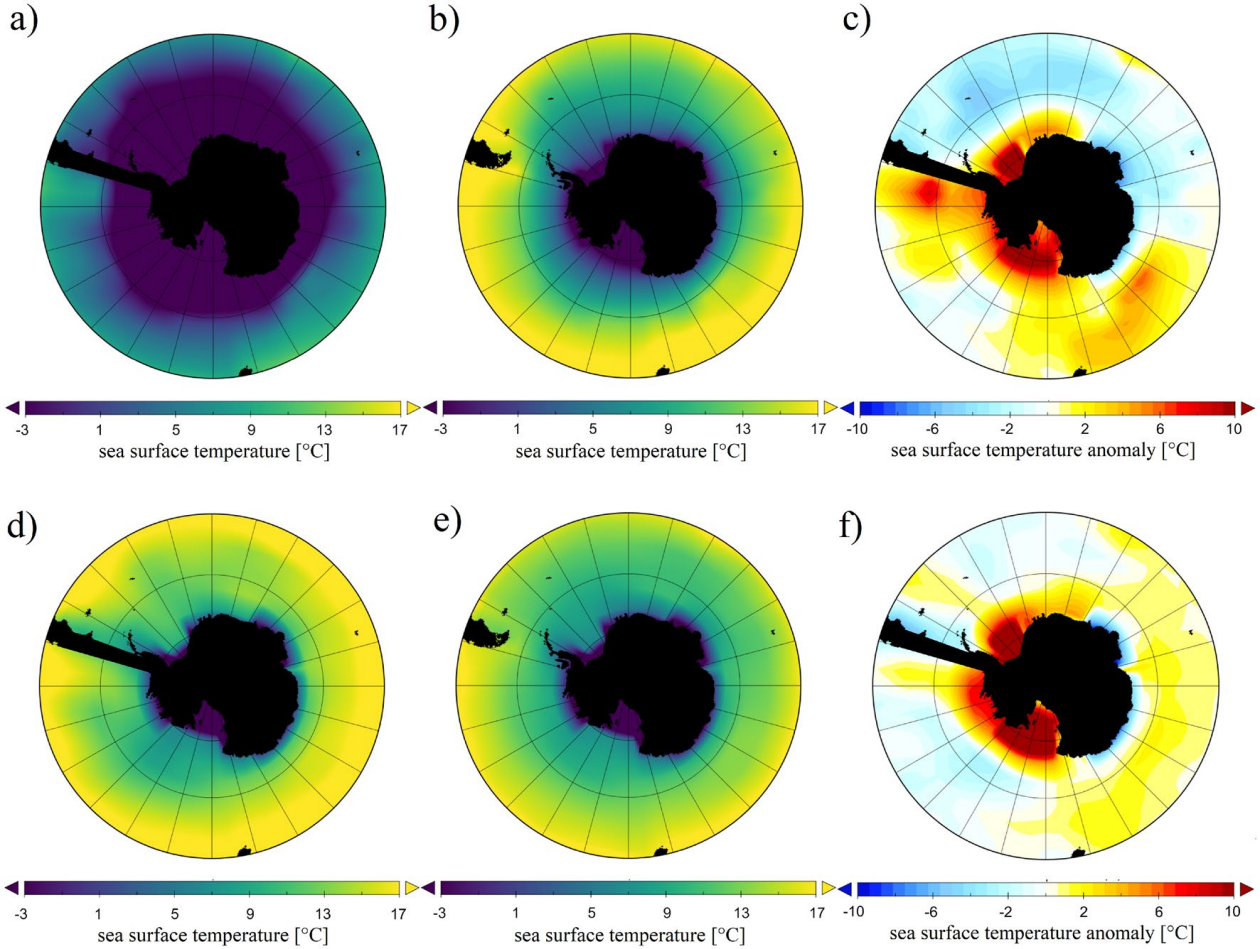
Mean surface temperature fields from the GCM: for “ice OFF” closed (**a**), open (**b**), closed “anomaly” (**c**) cases, and “ice ON” closed (**d**), open (**e**), closed “anomaly” (**f**) cases. “Closed anomaly” here (panels **c** and **f**) means that the 200-year zonal averages are subtracted from the “closed” maps. The figure was created using Panoply 4 developed at the NASA Goddard Institute for Space Studies (freely available and downloadable at https://www.giss.nasa.gov/tools/panoply/).

Instead of implementing the realistic paleogeographic shorelines and the (mostly unknown) paleobathymetry of the Eocene epoch, the “closed” configuration was modeled simply by a full-depth meridional barrier (“dam”) in the Drake Passage connecting the tip of South America and the Antarctic peninsula, while otherwise keeping the present-day continental arrangement unchanged, as sketched in Fig. 5. In the zonal direction the barrier spanned two grid cells (i.e. a domain of $11.2^\circ$) on PlaSim’s grid with a resolution of T21. Each simulation covered a 1000-year time frame, of which the last, already quasi-stationary 200-year interval was evaluated in terms of annually averaged surface temperature fields both in the “closed” and “open” configurations (no barrier, modern topography). The model runs were conducted using an estimate^25^ for the Late Eocene–Early Oligocene CO$_2$ level, 750 ppm.

As an adjustable setting, the so-called ice module of PlaSim could be switched on or off. When active, the module computes the extent and thickness of sea ice dynamically as the temperature field changes. When turned off, however, even when the water temperature decreases below freezing point in a given grid cell, the formation of ice shelves is inhibited and, hence, so are the resulting effects on the system (most notably, the ice-albedo feedback). The latter setting therefore approximates the laboratory experiment where sea ice dynamics is also absent. Fig. 5 shows the sea surface temperature fields in “closed” and “open” simulations for both ice module settings (ice OFF and ice ON in the top and bottom rows, respectively). For the “closed” cases the “anomaly” maps were also computed by averaging over the aforementioned 200-year period and subtracting the zonal means in each grid cell. The resulting patterns are presented in paenls c) (ice OFF setting) and f) (ice ON setting). This anomaly map of the ice OFF case indeed resembles the one seen in the experimental model (Fig. 2a) with pronounced warm and cold anomalies at the Pacific and Atlantic sides of the barrier, respectively.

**Figure 6:**
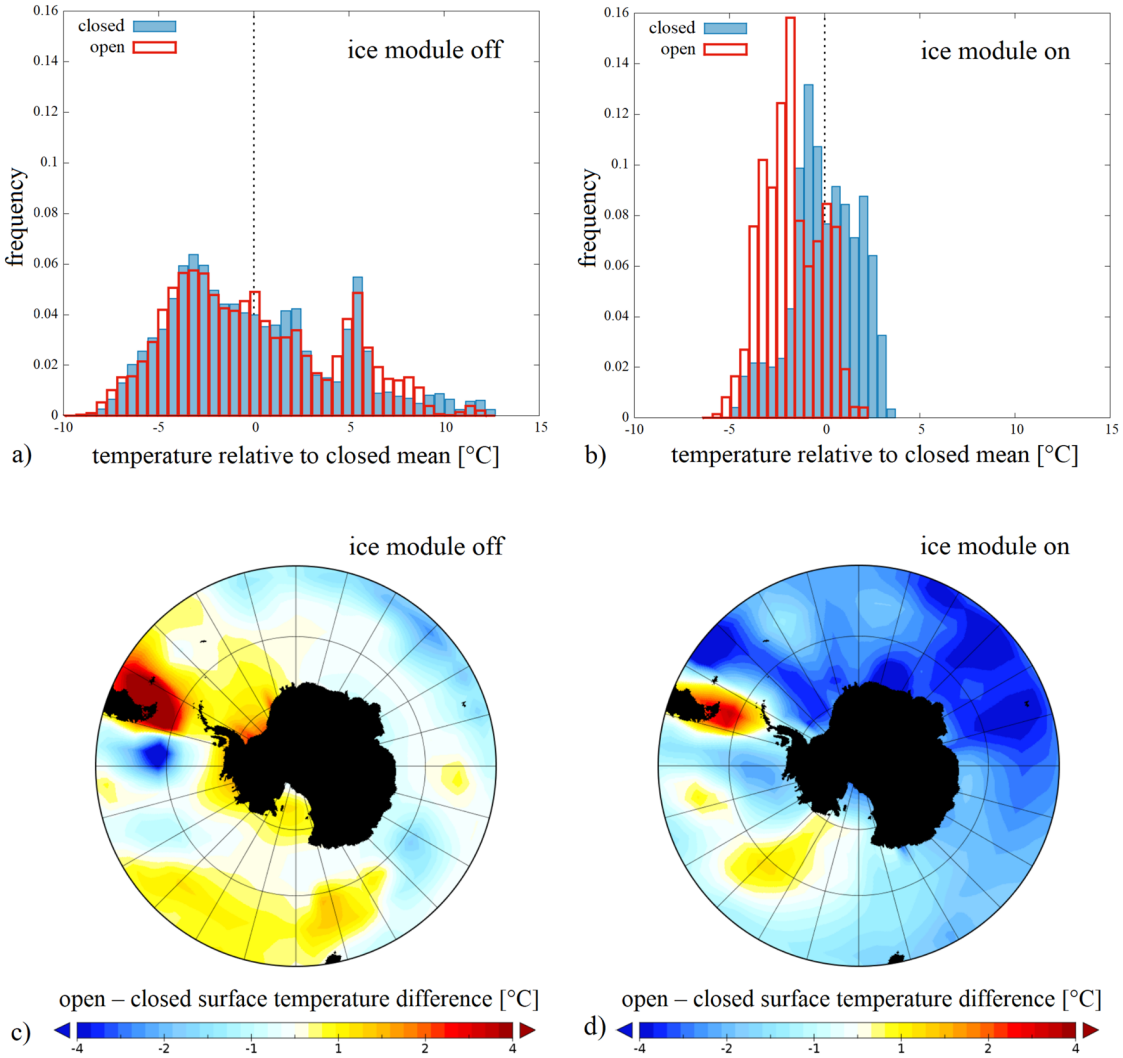
Surface temperature anomalies in the GCM. a, b, Histograms of the sea surface temperature anomaly field of the domain of the Southern Ocean (between latitude $55^\circ$S and the Antarctic circle) acquired from the annually averaged temperature fields of 200 subsequent years in both the “closed” (blue) and the “open” (red) configurations. The anomalies here are given as the deviation from the mean of the “closed” distributions (hence both “closed” histograms are exactly zero-centered). Panels (**a**) and (**b**) correspond to the inactive and active sea ice dynamics presets, respectively. (**c**, **d**) Temporally averaged maps of sea surface temperature change, i.e. the difference between temporal mean temperatures of the two barrier configurations $\langle T_{\mathrm{open}}\rangle -\langle T_{\mathrm{closed}}\rangle$ corresponding to the inactive (**c**) and active (**d**) sea ice dynamics presets. Panels (**a**) and (**b**) were created using Gnuplot version 5.2, Release 5.2.8 (freely available and downloadable at http://www.gnuplot.info/), panels (**c**) and (**d**) were created using Panoply 4 developed at the NASA Goddard Institute for Space Studies (freely available and downloadable at https://www.giss.nasa.gov/tools/panoply/).

Panels (a) and (b) of Fig. 6 show histograms of annually averaged surface temperature fields in the domain of the Southern Ocean (i.e. between latitude $55^\circ$S and the Antarctic circle) as acquired from the aforementioned 200-year-long series of quasi-stationary data. The blue and red graphs represent the distributions from the “closed” and the “open” configurations, respectively. The temperature scale was adjusted so that the mean of the “closed” record is subtracted from all measured values, thus the “closed” histograms are centered to zero in both panels. In panel (a) the GCM’s ice module was turned off, whereas in panel (b) it was active. The most striking feature of the simulations with inactive ice module (Fig. 6a) is that the histograms remain practically identical in the “open” and “closed” cases. However, when the ice module is active in the model (Fig. 6b), the “open” configuration yields markedly lower surface temperatures in the Southern Ocean, in qualitative agreement with the EOT paleotemperature records.

The spatial patterns associated with the (imaginary) opening event’s impact on the time-averaged surface temperatures of the Southern Ocean and Antarctica are demonstrated in the maps of Figs. 6c and d. The difference between the average values from the “open” and “closed” configuration $\langle T_{\mathrm{open}} \rangle - \langle T_{\mathrm{closed}} \rangle$ is plotted in both settings of the sea ice module. In the absence of sea ice dynamics (panel c) the domain up to ca. $70^\circ$S exhibits markedly higher temperatures in the “open” configuration than in the “closed” one. In the fully ocean-covered latitudes (North of the $70^\circ$S circle) the difference between the “open” and “closed” cases is negligible in terms of zonal means (cf. Fig. 6a). Averaging over the investigated domain (or the entire globe), therefore a slight temperature increase can be observed in the “open” configuration relative to the “closed” one. With active ice module (panel d), however, the “open” configuration clearly produces lower surface temperatures at all latitudes of the Southern Ocean, consistently with the EOT records.

To summarize, sea ice dynamics—most notably ice-albedo feedback—appears to play a key role in the chain of mechanisms that connect the opening of the Drake Passage and the overall decrease of temperature. Without sea ice dynamics the opening *per se* does not cause such cooling, in agreement with the findings from the laboratory experiment.

## Discussion

Our results indicate that the classic interpretation of the EOT global cooling, and the narrative of Antarctic glaciation in particular, needs to be reconsidered. The findings from a conceptual laboratory model as well as numerical simulations in a GCM suggest that under constant thermal forcing conditions the opening of a meridional barrier does not necessarily yield an overall decrease of surface temperatures. Moreover, it may even have the opposite effect—a slight warming in the investigated region—if sea ice formation is inhibited. If, however, the conditions are such that they allow for ice coverage to be present in the “closed” and “open” configurations alike, then indeed—in line with the paleoclimate record—the South polar region gets markedly colder in the “open” case than in the “closed” one.

Earlier numerical simulations^26^ also indicated that closing the present-day Drake Passage alone would not necessarily affect the mean SST in the Southern Ocean significantly. Instead, the observed differences between the closed and open configurations were found to be very sensitive to the CO_2_ concentration and, hence, the sea ice cover in the region. Moreover, another work using a fully coupled atmosphere-ocean model with a mid-Oligocene geography^27^ suggests that the development of ACC appears to be more of a consequence than a driver of the global cooling, and as such it rather acted as a feedback mechanism in the EOT. More recent numerical studies^28,29^ also emphasized the amplifying role of the ice-albedo feedback in the process, in agreement with our findings.

The question arises, however, of why we observe increased water surface temperatures in the laboratory experiment after the opening, whereas the Southern Ocean’s mean surface temperature remains practically unaffected by changing the configuration of the Drake Passage in the ice-free (meaning: “ice off” setting, sea ice is not simulated) GCM simulations (Fig. 6). First, it is important to realize that the thermal boundary conditions are somewhat different in the two settings. In the laboratory, the temperature of the middle cylinder (representing Antarctica) is kept at a fixed, prescribed value, whereas in the GCM dynamic heat exchange takes place between Antarctica and the ocean. As the differential map of Fig. 6c shows (for the “ice off” setting), certain partially land-covered latitudes (i.e. approximately those south of 70°S) indeed exhibit higher temperatures in the open configuration, which may well be the consequence of heat release from the sea surface to the atmosphere. Therefore, the experimental and numerical results, despite their obvious differences, reassuringly point to the same direction in the sense that no significant decrease of water temperatures could be detected in the absence of sea ice dynamics.

Our laboratory experiment is a radically simplified, abstract representation of the Southern Ocean. The setting does not capture wind stress or interior deep-ocean mixing, just to name two crucial factors that propel and shape the present-day ACC. Beta effect would also play a key role in a more realistic closed configuration, as the northward flow at the closed passage would likely form a western boundary current. Nor does our laboratory model involve the effects of other continental boundaries and heat and material exchange with the ocean gyres. Nevertheless, the lessons learned here on the heat transfer of quasi-geostrophic flows in the two most basic topological configurations are relevant and insightful. Removing the barrier allows the formation of an ACC-like meandering circumpolar jet, and blocks the development of a mean zonal pressure gradient, thus reducing meridional heat transport. In this “open” setting the mainly eddy-driven remaining overturning is expected to yield increased vertical temperature contrast in the system, which manifests in the increase of surface temperatures.

These findings, even though they may seem counterintuitive, provide circumstantial evidence in favor of the assumption that the Antarctic coastal waters may already have been, at least partially, ice-covered before the opening of the Drake Passage^5^. With sea ice already present at the continent, the opening could indeed catalyze further ice formation (presumably via ice-albedo feedback) and could eventually lead to the thermal isolation of Antarctica and the strengthening of the ACC. However, in the hypothetical case of an initially sea ice-free Antarctica the continent could have become even warmer following the opening, a scenario not indicated by paleotemperature reconstructions. The proposed story line is consistent with the interpretation of DeConto and Pollard^6^, who argued that the falling atmospheric CO_2_ level was the primary cause for the glaciation of Antarctica, and consequently, this event could possibly predate the opening of the gateway.

## Methods

### Laboratory experiments

The desired hydrodynamical similarity of the flow in the experiment to the actual ocean circulation requires certain nondimensional numbers (similarity parameters) to match, at least to the order-of-magnitude. In case of thermally driven rotating flows that are studied here, the most important similarity parameter is the thermal Rossby number (also known as Hide number) $Ro_T$, which has the form

1 $$\begin{aligned} {\text{Ro}}_T=\frac{\alpha g H \Delta T_r}{\Omega ^2 L^2}, \end{aligned}$$

where $g = 9.81$ m/s$^2$ is the gravitational acceleration, $\alpha = 2.07\times 10^{-4}$ 1/$^\circ$C represents the volumetric thermal expansion coefficient of the fluid, *L* is the characteristic horizontal length-scale (e.g. basin size), *H* denotes the fluid depth, $\Omega$ is the rotation rate (angular velocity) of the system and $\Delta T_r$ is the lateral (‘meridional’) temperature difference. The other nondimensional parameter of key relevance for the present configuration is the Taylor number *Ta* which quantifies the relative importance of viscous effects:

2 $$\begin{aligned} Ta=\frac{4\Omega ^2 L^5}{\nu ^2 H}, \end{aligned}$$

where $\nu = 1.004 \times 10^{-6}$ m$^2$/s denotes the kinematic viscosity of the fluid. The values of these parameters in large-scale ocean currents – with a horizontal length scale comparable to the radius of Earth – are typically of the orders of $\mathcal {O}(Ro_T)\le 10^{-2}$ and $\mathcal {O}(Ta)\ge 10^8$.

For our experiments two differentially heated rotating annuli were used, one located at the von Karman Laboratory of the Eötvös University (Budapest, Hungary) and another at the Fluid Centre of the Brandenburg University of Technology (Cottbus, Germany). The geometrical parameters of the Budapest tank are as follows: the radius of the inner (cooled) cylinder is $R_1 = 4.5$ cm, the radius of the outer (heated) cylindrical sidewall is $R_2 = 15$ cm and the applied water depth was $H = 5$ cm. The values of the same parameters in the Cottbus setup are $R_1 = 4.5$ cm, $R_2 = 12$ cm, $H = 5$ cm. The rotation rate of the experimental tank was set to a constant angular velocity $\Omega = 2.0$ rad/s in all cases. The blocking barriers were made of wood and acrylic and had a width of $R_2 - R_1$, a thickness of $d = 0.5$ cm and blocked the flow in the full depth. The temperature difference $\Delta T_r$ between the sidewalls that is to be kept constant throughout the experiments is regulated by means of Laude ProLine heating and cooling thermostats.

Seven of the experiment runs were conducted in the Budapest tank, which is equipped with a $4\times 16$ pixel Melexis thermal imaging sensor, mounted above the tank as sketched in Fig. 1. The sensor is characterized by a narrow radial “footprint” with an effective field of view of $16^\circ \times 60^\circ$ spanning from the inner to the outer cylindrical sidewall. 12 pixels in the radial (“meridional”) domain of the footprint is occupied by the free water surface of the annular gap. As the tank rotates underneath, the sensor thus scans the water surface temperature (WST) field with a sampling rate of 10 Hz. It is to be noted, that the penetration depth of the applied wavelength range (i.e. $7.5-14\mu$m) into water is less than a millimeter. The thermographic image of Fig. 2a was obtained by a co-rotating InfraTec VarioCam infrared camera mounted above the setup, operating in the same spectral range as the Melexis sensor.

The temperature setpoints and the corresponding values of $Ro_T$ and *Ta*—calculated with $L=R_2-R_1$ as the horizontal scale—are listed in Table 1. All the experiment runs started with a closed barrier and after at least 1800 revolutions (1.5 hours) the barrier was removed instantaneously by pulling it upward manually. Instead of running separate experiments with “open” and “closed” configurations, we have chosen to apply this dynamic procedure in order to ensure that the external conditions, affected by uncontrollable variations of the laboratory environment, stay precisely the same in the “open” and “closed” cases.

The studied parameter range falls in the same geostrophic turbulent dynamical regime as the aforementioned values ($\mathcal {O}(Ro_T)\le 10^{-2}$ and $\mathcal {O}(Ta)\ge 10^8$) representing ACC, see e.g. the regime diagram in Ref.^19^, therefore the fact that the nondimensional numbers do not match precisely are not relevant from a qualitative point of view.

The control experiment to compare the temperature distributions close to the bottom—inaccessible for the infrared sensors – and near the surface (see Fig. 3c,d) was conducted in the Cottbus tank, using Ahlborn ALMEMO NiCr sensors with a relative resolution of $0.05^{\circ }$C and a sampling rate of 1 Hz. The sensors for the near-surface and near-bottom temperatures were both fixed onto the same co-rotating mast above the free surface of the rotating annulus, and penetrated 6 mm-deep into the water surface and 5 mm from the bottom, respectively. The data were streamed in real-time through the co-rotating UHF module ALMEMO 8590-9.

### The Planet Simulator (PlaSim) GCM

In the present study the climate model, Planet Simulator (PlaSim)^22^ is used to simulate Earth’s climate. PlaSim was developed to understand the main physical processes in climate dynamics. In previous studies (see, e.g.,^30–32^) it proved to be an appropriate numerical tool to investigate possible behaviour of the climate system on global scale.

For our purposes, we make use of the same atmospheric setup as in Ref.^30^, i.e., the horizontal resolution of the simulations is T21, which yields a grid of approximately 5.6° × 5.6°. As a non-standard aspect of our model setup, the PlaSim atmosphere is coupled to a large scale geostrophic (LSG) ocean (original name: The Hamburg Large Scale Geostrophic Ocean General Circulation Model (Cycle 1)^24^. The atmospheric dynamics are described by primitive equations that represent conservation laws, thermodynamics and the hydrostatic approximation. Through parameterization, the model accounts for numerous unresolved processes, including sea ice formation, which is a key feature in our study. The so-called sea ice module is based on the zero layer model of Ref.^33^. This model computes the thickness of the sea ice from the thermodynamic balances at the top and the bottom of the sea ice. The zero layer model assumes the temperature gradient in the ice to be linear and eliminates the capacity of the ice to store heat. For each marine cell on the grid, sea ice is allowed to form when the surface temperature drops below 271.25 K (–1.90 °C). If a grid cell is covered by sea ice, snowfall is accumulated on top of the ice. Snow is converted to sea ice if there is sufficient snow to suppress the ice/snow interface below the sea level. The typical sea ice thickness for the fully ice-covered sea is around 1 meter.

The LSG ocean model is based on the observations that for large scale ocean circulation models designed for climate studies, the relevant characteristic spatial scales are large compared to the internal Rossby radius throughout most of the ocean. At the same time the characteristic time scales are large compared with the periods of gravity modes and barotropic Rossby wave modes. The LSG ocean model was developed by Maier-Reimer and Mikolajewicz in early 1990s^24^. This LSG ocean model was originally proposed by^34^, and is described more fully by^35^, and it has been used in a number of climate and paleoclimate studies (see, e.g.,^35–39^). In Maier-Reimer et al. 1993 the LSG ocean model was investigated in details. It has been showed that the simulated mean ocean circulation for appropriately chosen surface forcing fields adequately and realistically reproduces the principal water mass properties, residence times, and large-scale transport properties of the observed ocean circulation within the constraints of the model resolution. We used the default resolution of 3.5 x 3.5 degrees and 22 non-equidistant vertical layers along with a realistic present-day bathymetry. The typical maximum basin depth is 5500 meters. We mention that the LSG model was also applied previously to investigate the role of the closed Drake Passage in ocean dynamics^38^. In our model setup the original LSG model is modified, the “closed” configuration was modelled simply by a full-depth meridional barrier (“dam”) in the Drake Passage connecting the tip of South America and the Antarctic Peninsula, otherwise keeping the present-day continental arrangement unchanged. The initial state and other properties of the LSG model ocean are the default ones^24^. The LSG ocean is spun up with present geography and bottom topography for a period of 10000 years to reach steady state. After the spin-up we compute a 1000 year long period to investigate the impact of the closed/open Drake Passage.

## Supplementary Information

Supplementary Information.

## Data Availability

Measured raw data are available upon request from the corresponding author (M. Vincze).
