# Supplementary material for: The Drake Passage opening from an experimental fluid dynamics point of view
Source: Sci Rep. 2021 Oct 7;11:19951. doi: 10.1038/s41598-021-99123-0 (PMC8497466; doi:10.1038/s41598-021-99123-0)
Supplement: Supplementary file 1 — Supplementary Information. [file 41598_2021_99123_MOESM1_ESM.pdf]

**Supplementary information for:**

# The Drake Passage opening from an experimental fluid dynamics point of view

Miklós Vincze, Tamás Bozóki, Mátyás Herein, Ion Dan Borcia, Uwe Harlander, Attila Horicsányi, Anita Nyerges, Costanza Rodda, András Pál, József Pálfy

In what follows, we provide a brief summary about the background of our numerical simulations.

**Ocean dynamics (LSG OGCM)**

We make a use of the LSG ocean model (full name: The Hamburg Large Scale Geostrophic Ocean General Circulation Model (Cycle 1)) developed by Maier-Reimer and Mikolajewicz in early 90s[1].

This LSG ocean model was originally proposed by Hasselmann[2], is described more fully by Maier-Reimer et al.[3], and has been used in a number of climate and paleoclimate studies[4-8] . In ref. [3] the LSG ocean model was investigated in detail. It has been shown that the simulated mean ocean circulation for appropriately chosen surface forcing fields reproduces well the principal water mass properties, residence times, and large-scale transport properties of the observed ocean circulation quite realistically within the constraints of the model resolution.

We used in this study the default resolution with 3.5 x 3.5 degrees and 22 vertical layers along with a present realistic bathymetry. We mention that the LSG model was also applied previously to investigate the role of the closed Drake Passage in ocean dynamics[7].

The motivation for the use of LSG-OGCM is based on the observation that for large-scale ocean circulation, the characteristic spatial scales are large compared with the internal Rossby radius. In addition, the characteristic timescales are large compared with the periods of gravity modes and barotropic Rossby waves. Since it is generally believed that these waves are not important for climate research studies, they have been filtered out of the model.

The nonlinear advection of momentum has been excluded in the set of primitive equations, and the equations are integrated with an implicit method which permits a time step of 30 days. This makes the model numerically efficient and permits integrations over thousands of years. The free surface is treated prognostically, without invoking the rigid lid approximation. The numerical scheme is unconditionally stable and can be applied uniformly to the entire globe.

Convective overturning is introduced whenever the stratification becomes unstable. The water column is stabilized with the minimum mixing compatible with the conservation of heat and salt: water in the thinnest layer is exchanged with an equal volume of water from the thickest layer. The water newly injected into this cell is then completely mixed with the residual water. This algorithm is applied successively to all pairs of layers, beginning at the top.

**Governing equations**

The baroclinic velocity field can be derived diagnostically from the given density field through local geostrophy. Equally the barotropic velocity field and surface elevation can be determined diagnostically for a given density field as the equilibrium barotropic response of the prescribed surface-stress forcing and bottom torque. The only prognostic equations are thus the remaining advection equations for temperature and salinity.

Applying these conditions, the full equations for the ocean can be strongly simplified. Using the usual hydrostatic and Boussinesque approximations, the complete set of prognostic equations is given by;

$$\frac{\partial \underline{u}}{\partial t} - f\,\underline{u} + \nabla p = \underline{q} \qquad \text{(S1)}$$

$$\frac{\partial \zeta}{\partial t} - w_0 = q_\zeta \approx 0 \qquad \text{(S2)}$$

$$\frac{\partial T}{\partial t} + \left( (\underline{u}\,\nabla) + w\frac{\partial}{\partial z} \right) T = q_T \qquad \text{(S3)}$$

$$\frac{\partial S}{\partial t} + \left( (\underline{u}\,\nabla) + w\frac{\partial}{\partial z} \right) S = q_S \qquad \text{(S4),}$$

where $\underline{u}$ is the horizontal velocity, ζ the surface elevation, T temperature, S salinity, respectively. f is the Coriolis parameter, w is the vertical velocity, $w_0$ is the value of w at the surface (z=ζ; z is vertical coordinate)). The forcings on the right-hand side of (S1), (S3) and (S4) represent the sum of turbulent eddy transport. The forcing term in (S2) represents the sum of the mass flux due to precipitation or evaporation, which is relatively small and it is neglected in this model. Eddies are incorporated into the forcing term ($\underline{q}$). The forcing term in (S1) is given by the anisotropic turbulent friction expression:

$$\underline{q} = \upsilon_V \frac{\partial^2 \underline{u}}{\partial z^2} + \upsilon_H \Delta \underline{u} \qquad \text{(S5).}$$

In (S5) $\upsilon_V$ and $\upsilon_H$ denote the vertical and horizontal eddy viscosity coefficients (the details can be found in ref. [2]).

**Atmospheric dynamics (PlaSim)**

The numerical model used here is PLASIM[9], a simplified GCM developed at the University of Hamburg. The primitive equations are solved by a spectral transform method. The model has a full set of physical parameterizations for unresolved processes. Parameterizations include long and shortwave radiation with interactive clouds, horizontal and vertical diffusion, boundary layer fluxes of latent and sensible heat. Stratiform precipitation is generated in supersaturated states, and the Kuo scheme is used for deep moist convection, while shallow cumulus convection is parameterized by means of vertical diffusion. The description of PlaSim's atmospheric dynamics (e.g., radiation transfer) is beyond the frame of this writing, thus please see the Reference Manual that is freely available together with the code at:

https://www.mi.unihamburg.de/en/arbeitsgruppen/theoretischemeteorologie/modelle/plasim.html

for a detailed and referenced description of the PlaSim framework.

**Sea ice dynamics (PlaSim)**

The only available dynamical ice module of PlaSim is the sea ice model, however non-dynamical (stationary) land ice (glacier landforms, e.g., permanent ice sheets) can be prescribed in the model, as well. For each marine cell on the grid, sea ice is allowed to form when the surface temperature is below 271.25 K (−1.90 ◦C). The sea ice model is based on the zero layer model of Semtner[10]. This model computes the thickness of the sea ice from the thermodynamic balances at the top and the bottom of the sea ice. The zero layer assumes the temperature gradient in the ice to be linear and eliminates the capacity of the ice to store heat. If the surface temperature of open ocean water is below the freezing point, sea ice is formed. If a grid point is covered by sea ice, snowfall is accumulated on top of the ice.

Snow is converted to sea ice if there is sufficient snow to suppress the ice/snow interface below the sea level. The typical sea ice thickness for the fully ice covered sea ranges between 1-10 m.

**Basic sea ice equations**

In the presence of sea ice, the total heat flux (Q) is defined as

$$Q = Q_a + Q_c + Q_o + Q^* \qquad \text{(S6)}$$

where $Q_a$ is the atmospheric heat flux, $Q_c$ is the conductive heat flux through sea ice, $Q_o$ is the oceanic heat flux and $Q^*$ is the flux correction. The details can be found in refs. [10,11].

**Reaching quasi-stationarity**

The characteristic time scale of the deep ocean may be at least hundreds or even thousands of years. Therefore, it is necessary to run climate models for thousands of years to reach a steady state (or quasi-stationary state). In our case, such a state is reached as follows. First, the LSG (Large Scale Geostrophic) ocean module is spun up with present-day geography and bottom topography for a period of 10 000 years. After this spin-up we simulate a 1000 year-long period to investigate the impact of the Drake Passage configuration (open or closed). It is to be emphasized that unlike in the laboratory experiment we do not model a time-dependent opening of the Passage, but compare separate quasi-stationary runs with the two configurations.

The stationarity of the runs reported in the manuscript was evaluated, and it was found that the system reaches a quasi-stationary state within several hundred years after the initialization of the 1000 year-long run. This quasi-stationarity is illustrated by the figure below (Fig. S1) showing the time evolution of the global mean surface temperature in the closed configuration with active sea ice dynamics. Apparently, after the initialization there is a pronounced transient period for several hundreds of years, after which the simulation can be considered quasi-stationary. If we consider the last 200 years only (Fig.S2), it is clearly visible that quasi-stationarity is reached.

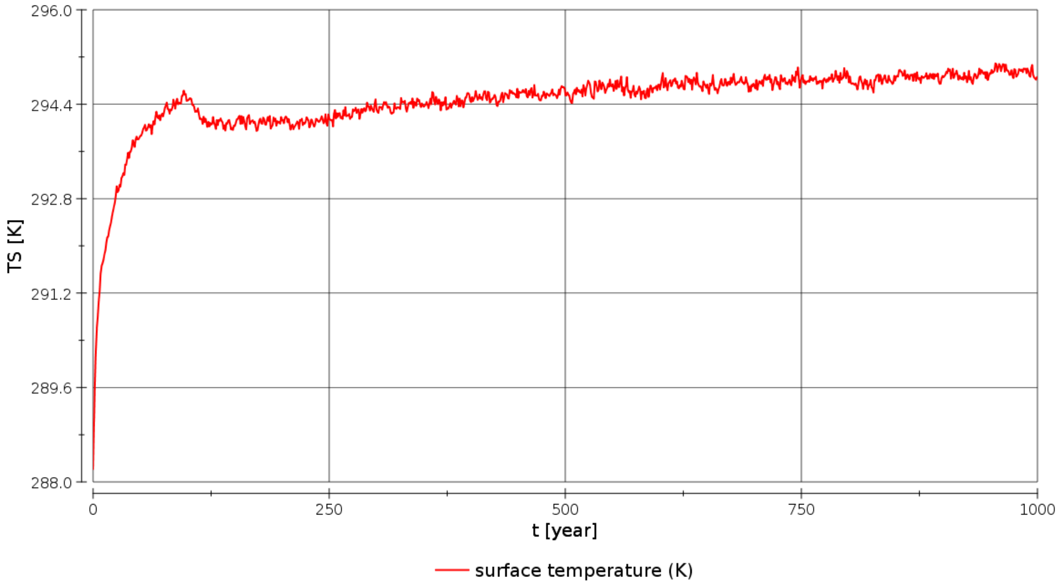


**Fig. S1:** Time evolution of global mean surface temperature (TS) in the closed configuration

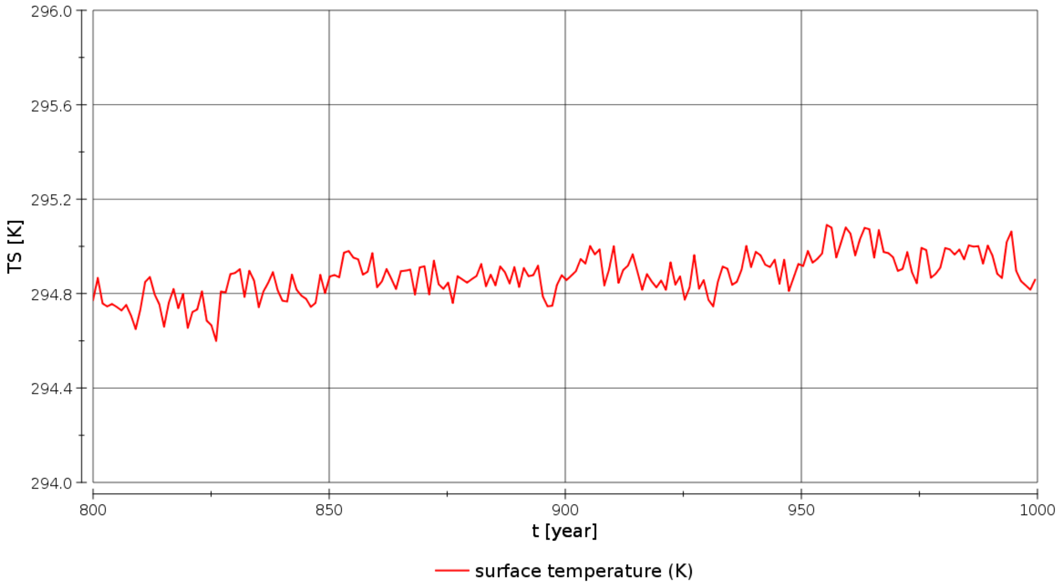


**Fig. S2:** Time evolution of global mean surface temperature (TS) in the closed configuration, last 200 hundred years of the simulation.

**Limitations of the PlaSim-LSG model**

The climate model PlaSim was developed to capture the large-scale dynamics of the climate system. Obviously being a mid-complexity climate model, its outcomes are referred on synoptic meteorological scale only, which naturally designates its limitations.

Even if indeed not competitive with state of-the-art GCMs, PlaSim produces a fairly realistic present climate on a synoptic scale. It is representative of the class of complex numerical models applied for climate modelling, in particular understanding geophysical fluid dynamical problems[9,12-14].

In this study PlaSim is coupled to the LSG-OGCM ocean model[6].The limitations of LSG-OGCM relies on the lack of complex turbulent dynamics. Nonlinear terms in the Navier-Stokes equations are neglected, furthermore the hydrostatic and the Boussinesq approximations are applied[6]. Turbulent motions are parameterized by means of a vertical oceanic diffusion coefficient, which is a rather simple function of the vertical coordinate[15]. As a result, the modelling results can be valid strictly on synoptic scale only, mirroring the general large-scale behaviour of the climate system and they are not suitable to create accurate climate projections.

**References**


1. Maier-Reimer, E., & Mikolajewicz, U. (1991). The Hamburg Large Scale Geostrophic Ocean General Circulation Model (Cycle 1). Technical Report / Deutsches Klimarechenzentrum, 2.
2. Hasselmann, K., An ocean model for climate variability studies. 1982. Prog., Oceanogr.,**1** 1, 69-92.
3. Maier-Reimer, E., U. Mikolajewicz, and K. Hasselmann, 1993: Mean Circulation of the Hamburg LSG OGCM and Its Sensitivity to the Thermohaline Surface Forcing. J. Phys. Oceanogr., **23**, 731–757, https://doi.org/10.1175/1520-0485(1993)023<0731:MCOTHL>2.0.CO;2.
4. Maier-Reimer, E., U. Mikolajewicz, and T. J. Crowley, 1990. Ocean general circulation model sensitivity experiment with an open central American isthmus, Paleoceanography, **5**, 349-366.
5. Mikolajewicz, U., B. D. Santer, and E. Maier-Reimer, 1990. Ocean response to greenhouse warming, Nature, **345**, 589-593.
6. Maier-Reimer, E. 1993. The driving force of brine rejection on the deep-water formation in the Hamburg LSG OGCM. In Peltier, W. R., ed. Ice in the climate system. Berlin, etc., Springer-Verlag, 211–216. (NATO ASI Series I: Global Environmental Change 12.)
7. Mikolajewicz, U., Maier-Reimer, E., Crowley, T. J., and Kim, K.-Y. (1993), Effect of Drake and Panamanian Gateways on the circulation of an ocean model, Paleoceanography**, 8( 4)**, 409– 426, doi:10.1029/93PA00893.
8. Drijfhout, S. S., Maier-Reimer, E., and Mikolajewicz, U. (1996), Tracing the conveyor belt in the Hamburg large-scale geostrophic ocean general circulation model, J. Geophys. Res., **101**( C10), 22563– 22575, doi:10.1029/96JC02162.
9. Fraedrich, K., Jansen, H., Kirk, E., Luksch, U. & Lunkeit, F. (2005) The Planet Simulator: Towards a user friendly model. Meteorol. Z. **14**, 299–304.
10. Semtner, A. J. Jr., A Model for the Thermodynamic Growth of Sea Ice in Numerical Investigations of Climate, J. Physic. Oceanogr., **3**, 379–389, 1976.
11. Lunkeit, F. et al. (2011). Planet Simulator Reference Manual Version 16 (Meteorological Institute, University of Hamburg). Hamburg.

12. Kleidon, A., K. Fraedrich, E. Kirk, and F. Lunkeit, (2006). Maximum entropy production and the strength of boundary layer exchange in an atmospheric general circulation model. Geophys. Res. Letters, 33, L06706
13. Holden, P.B., N.R. Edwards, P.H. Garthwaite, K. Fraedrich, F. Lunkeit, E. Kirk, M. Labriet, A. Kanudia, and F. Babonneau, (2014). PLASIM-ENTSem: a spatiotemporal emulator of future climate change for impacts assessment. Geoscientific Model Development 7, 433-451.
14. Holden, P.B., N.R. Edwards, K. Fraedrich, E. Kirk, F. Lunkeit, and X. Zhun (2016). PLASIM-GENIE: a new intermediate complexity AOGCM. Geosci. Model Dev. 9, 3347-3361.
15. Bryan, K., Lewis, L. J.: A water mass model of the World Ocean (1979). J. Geophys. Res., 84(C5), 2503-2517, https://doi.org/10.1029/JC084iC05p02503.
